# Supplementary material for: Enzyme promiscuity shapes adaptation to novel growth substrates
Source: Mol Syst Biol. 2019 Apr 8;15(4):e8462. doi: 10.15252/msb.20188462 (PMC6452873; doi:10.15252/msb.20188462)
Supplement: Supplementary file 1 — Appendix [file MSB-15-e8462-s001.pdf]

Appendix

Table of contents

1. Appendix Tables S1: Non-native substrates considered for evolution experiments
2. Appendix Table S2: Laboratory evolution parameters of interest
3. Appendix Table S3: One week mutant growth test summary
4. Appendix Table S4: Growth Rate Analysis - AraC mutations Impact
5. Appendix Table S5: Sanger Sequencing Primers
6. Appendix Table S6: Mass spectrometry methods summary (Parameters and settings used for data acquisition on the Agilent 6550 model QTOFs)
7. Appendix Figure S1: Evolution growth rate trajectory curves
8. Appendix Figure S2: Contingency Appendix Table Showing agreement between computational predictions and experimental adaptation
9. Appendix Figure S3: Regions of duplication observed in D-2-deoxyribose evolution strains
10. Appendix Figure S4: D-2-deoxyribose pORTMAGE library growth experiments
11. Appendix Figure S5: YihS wild type and mutant substrate conversion
12. Appendix Figure S6: AraB Ribulokinase activity predicted to improve growth rate *in silico*
13. Appendix Figure S7: Optimization mutation analysis for m-tartrate experiments
14. Appendix Figure S8: Large Deletion observed in endpoint population evolved on D-2-deoxyribose
15. Appendix Figure S9: Deletion mutation analysis for the D-2-deoxyribose experiment
16. Appendix Figure S10: Optimization mutation analysis for D-lyxose Experiment 2
17. Appendix Figure S11: Sequence alignment of RbsK mutant and DeoK from pathogenic strains
18. Supporting Text
19. References

Appendix Table S1: Non-native substrates considered for evolution experiments

Carbon source	Identified Gene with Underground Activity	Evidence for growth-supporting[1] underground activity
Phenylgalactoside	*lacZ**	biochemical
D-Lyxose	*yihS**	biochemical
D-Tartrate	*fumA**, *fumB** (*fumC*)	biochemical
D-2-Deoxyribose	*rbsK**, *deoC*	biochemical
L-Glyceraldehyde	*fucO, glpK*	biochemical
D-Arabinose	*rbsK*	biochemical
Ethylene glycol	*fucO*	biochemical
5-Amino-4-oxopentanoate	*bioB*	biochemical
m-Tartrate	*dmlA**	inferred from gene overexpression phenotype and orthology information
Monomethyl Succinate	*ybfF**	inferred from gene overexpression phenotype and reaction chemistry

[1]In silico growth advantage of the underground activity was determined previously (Notebaart *et al*, 2014). For a subset of genes (marked with asterisks), Notebaart et al. also provided experimental evidence that overexpression of the specific gene conferred (or improved) growth on the carbon source. Note also that for two carbon sources, the growth-conferring enzyme and its underground reaction was inferred from an experimental overexpression screen and orthology / reaction chemistry data and no direct biochemical evidence is yet available in *E. coli* (see Notebaart et al. 2014 for details).

Appendix Table S2: Laboratory evolution parameters of interest

Experiment	Initial Growth Rate (hr^{-1})	Final Growth Rate (hr^{-1})	% of Final Growth Rate Gained during Static. Phase	# of Test Flasks tested during Weaning Phase	Generations in Weaning Phase	CCDs in Static Phase. ($x10^{11}$)	Generations in Static Phase
**D-Lyxose 1**	0.167 +/- 0.12	0.451 +/- 0.04	63	7	45	12.7	278
**D-Lyxose 2**	0.120 +/- 0.12	0.529 +/- 0.05	77	5	33	16.7	359
**D-2-Deoxyribose 1**	0.0253 +/- 0.041	0.431 +/- 0.03	94	18	71	16.1	422
**D-Arabinose 1**	0.187 +/- 0.12	0.418 +/- 0.02	55	6	36	13.7	310
**D-Arabinose 2**	0.208 +/- 0.10	0.558 +/- 0.01	63	5	37	15.6	353
**m-Tartrate 1**	0.101 +/- 0.04	0.278 +/- 0.02	64	2	16	14.1	354
**m-Tartrate 2**	0.0664 +/- 0.05	0.407 +/- 0.02	84	6	40	14.1	352
**Monomethyl Succinate 1**	0.360 +/- 0.02	0.353 +/- 0.01	*No significant increase	3	21	8.5	188
**Monomethyl Succinate 2**	0.250 +/- 0.03	0.316 +/- 0.01	21	4	29	7.5	182

Appendix Table S3: One week mutant growth test summary

Reconstructed single-/double mutations in BOP27	Growth Observed?
R315S yihS	Yes
R315C yihS	Yes
V314L yihS	Yes
I156S yihW	No
Δ2 bp (486-487) yihW	Yes
R315S yihS + V314L yihs	Yes
R315C yihS + V314L yihs	Yes
(619-624/993 nt) +TAATAG rbsR insertion	No
N20Y rbsK	No
D82Y fucR	Yes
S75R fucR	Yes
*244C fucR	Yes
A242T dmlA	Yes
C→T intergenic (-50/-53) dmlR ← / → dmlA	Yes
T→G intergenic (-35/-68) dmlR ← / → dmlA	Yes

Appendix Table S4: Growth Rate Analysis - AraC mutations Impact

Strain	Carbon Source	Mean Growth Rate (n=3)	Standard Deviation
BOP27(wild type)	D-arabinose	-	-
BOP27ΔfucK	D-arabinose	-	-
BOP27ΔaraBAD,yabI	D-arabinose	-	-
Exp1_weaned	D-arabinose	0.198	0.0155
Exp1_weanedΔfucK	D-arabinose	-	-
Exp1_weanedΔaraBAD,yabI	D-arabinose	0.237	0.0164
Exp1_optim	D-arabinose	0.411	0.0126
Exp1_optimΔfucK	D-arabinose	-	-
Exp1_optimΔaraBAD,yabI	D-arabinose	0.226	0.0125
Exp2_weaned	D-arabinose	0.200	0.0128
Exp2_weanedΔfucK	D-arabinose	-	-
Exp2_weanedΔaraBAD,yabI	D-arabinose	0.246	0.0140
Exp2_optim	D-arabinose	0.334	0.0188
Exp2_optimΔfucK	D-arabinose	-	-
Exp2_optimΔaraBAD,yabI	D-arabinose	0.267	0.00359
BOP27(wild type)	glycerol	0.441	0.0623
BOP27ΔfucK	glycerol	0.388	0.0298
BOP27ΔaraBAD,yabI	glycerol	0.366	0.037
Exp1_weaned	glycerol	0.354	0.0393
Exp1_weanedΔfucK	glycerol	0.363	0.0251
Exp1_weanedΔaraBAD,yabI	glycerol	0.415	0.065
Exp1_optim	glycerol	0.357	0.00992
Exp1_optimΔfucK	glycerol	0.423	0.00542
Exp1_optimΔaraBAD,yabI	glycerol	0.421	0.0488
Exp2_weaned	glycerol	0.335	0.0296
Exp2_weanedΔfucK	glycerol	0.464	0.00886
Exp2_weanedΔaraBAD,yabI	glycerol	0.416	0.0276
Exp2_optim	glycerol	0.473	0.0313
Exp2_optimΔfucK	glycerol	0.317	0.0179
Exp2_optimΔaraBAD,yabI	glycerol	0.453	0.021

Appendix Table S5: Sanger Sequencing Primers

Growth condition	Mutation regions to check	Forward (5'-3')	Reverse (5'-3')
D-lyxose	yihS	ACGACGTCGCCAGAAATAAT	ACCTTATTGTCCGCATCCAG
D-lyxose	yihW	CCGGGCGTTACTTAACCATA	GTTTGGCTATGTGGCAAGGT
D-2-deoxyribose	rbsK	ATGGCAAACTAGCAGCGACT	ATGCTGTCATCACCCGTACA
D-2-deoxyribose	rbsR	ATACCGAAGGCGATGAACAG	ACAGCCATAGCGTCATTTCC
D-arabinose	fucR	TGACCACGGAAGCTCTCTCT	TCAGGCACAGCTTGATGAAC
m-tartrate	dmlA, dmlR/dmlA	ACCACGTTTAGCGTTTGCTC	CCGAGAAAATCGAGCATCAT

Appendix Table S6: Mass spectrometry methods summary (Parameters and settings used for data acquisition on the Agilent 6550 model QTOFs)

Method	6550 Neg
Instrument	6550
Polarity	neg
Mode	MS
Gas temp (degrees C)	290
Injection volume (uL)	3
Drying gas (L/min)	11
Nebulizer pressure (psig)	30
Sheath gas temp (degrees C)	200
Sheath gas flow (L/min)	9
Capillary voltage (V)	3500
Nozzle voltage (V)	1000
Fragmentor (V)	175
Skimmer (V)	65
Peak-peak voltage applied to octopoles (V)	750
Min range (m/z)	20
Max range (m/z)	1200
Rate (spetra/s)	2
Transients/spectrum	4072
Collision energy (V)	0
Reference masses used for correction (m/z)	119.03632, 980.016375
Data storage format	Centroid
Data storage threshold (Abs)	200
Data storage threshold (%)	0.01

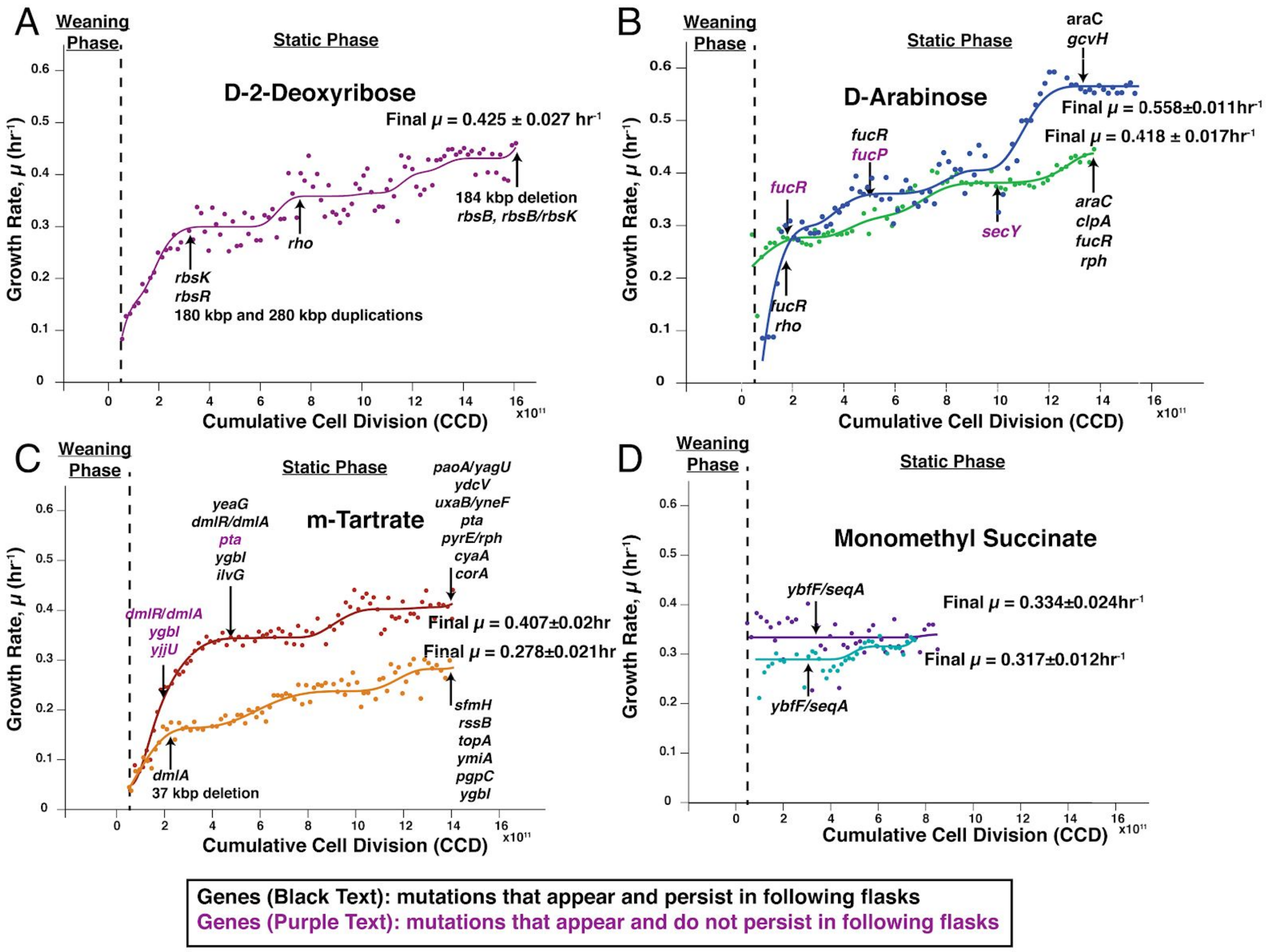

Appendix Figure S1: **Evolution growth rate trajectory curves.** The growth rate trajectory curves for the evolution on D-2-deoxyribose, D-arabinose, m-tartrate, and monomethyl succinate experiments are shown. Along the curves, time points at which samples were taken for whole-genome sequencing are highlighted by black arrows. Genes containing mutations following sequencing analysis of clones isolated at the sampled time points are also displayed.

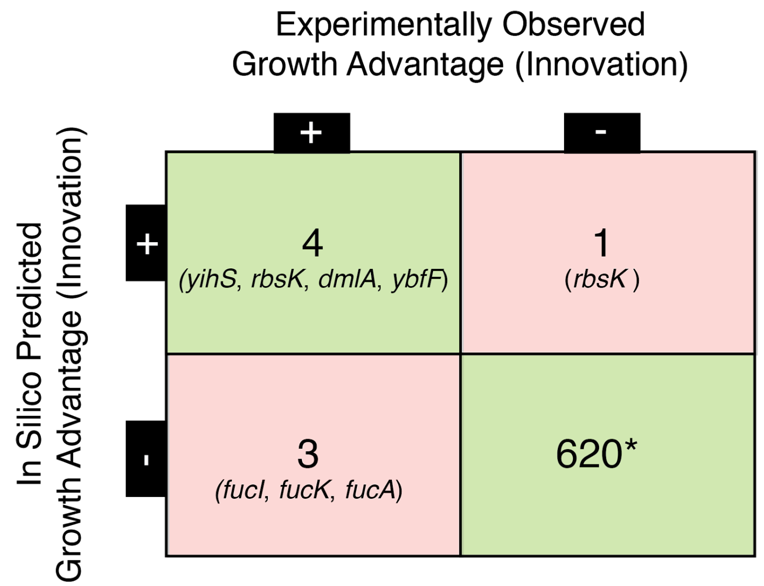

*Calculated: (125 genes promiscuous activity x 5 conditions) - 5

Appendix Figure S2: Contingency Appendix Table Showing agreement between computational predictions and experimental adaptation. There was significant agreement between the genes associated with predictions of promiscuity and the genes associated with experimental adaptation to the five non-native carbon sources. The contingency Appendix Table Shows that four genes (out of five) were correctly predicted to confer a growth advantage. There was disagreement in the case of growth on D-arabinose. The model predicted that promiscuous activity associated with *rbsK* would provide the growth advantage; however, the activities associated with *fucI*, *fucK*, and *fucA* were experimentally determined (LeBlanc & Mortlock, 1971) to enable adaptation to the non-native substrate. These three genes, *fucI*, *fucK*, and *fucA*, were indirectly determined to be conferring the growth advantage in the experiments presented in this paper due to the mutations (Table 1) observed in *fucR*, a DNA-binding transcriptional activator associated with regulating expression of *fucI*, *fucK*, and *fucA* (Podolny et al, 1999). Additional experimental evidence (Figure 3D) showed that the *fucK* gene was essential for growth of the evolved strains on D-arabinose. The number 620 comes from 125 genes previously shown to be associated with known promiscuous activity (Notebaart *et al*, 2014) multiplied by five growth conditions, subtracting five genes from the other boxes in the table; however, this number could be considerably larger based on unaccounted for promiscuous activities of other metabolic genes or other uncharacterized genes. The p-value associated with this table is smaller than 10^{-8}, Fisher's exact test.

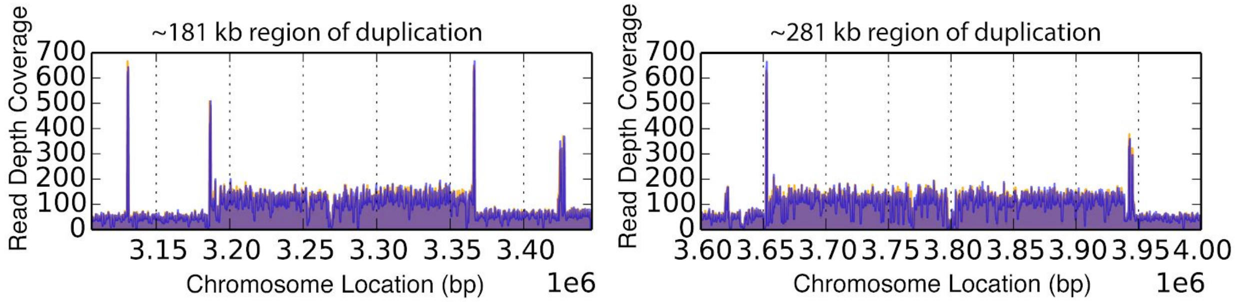

Appendix Figure S3: Regions of duplication observed in D-2-deoxyribose evolution strains. The large regions of genome amplification observed in the clone resequenced after initial adaptation to D-2-deoxyribose are displayed. These regions spanned 165 genes (*yqiG* - *yhcE*) and 262 genes (*yhiS* - *rbsK*), respectively.

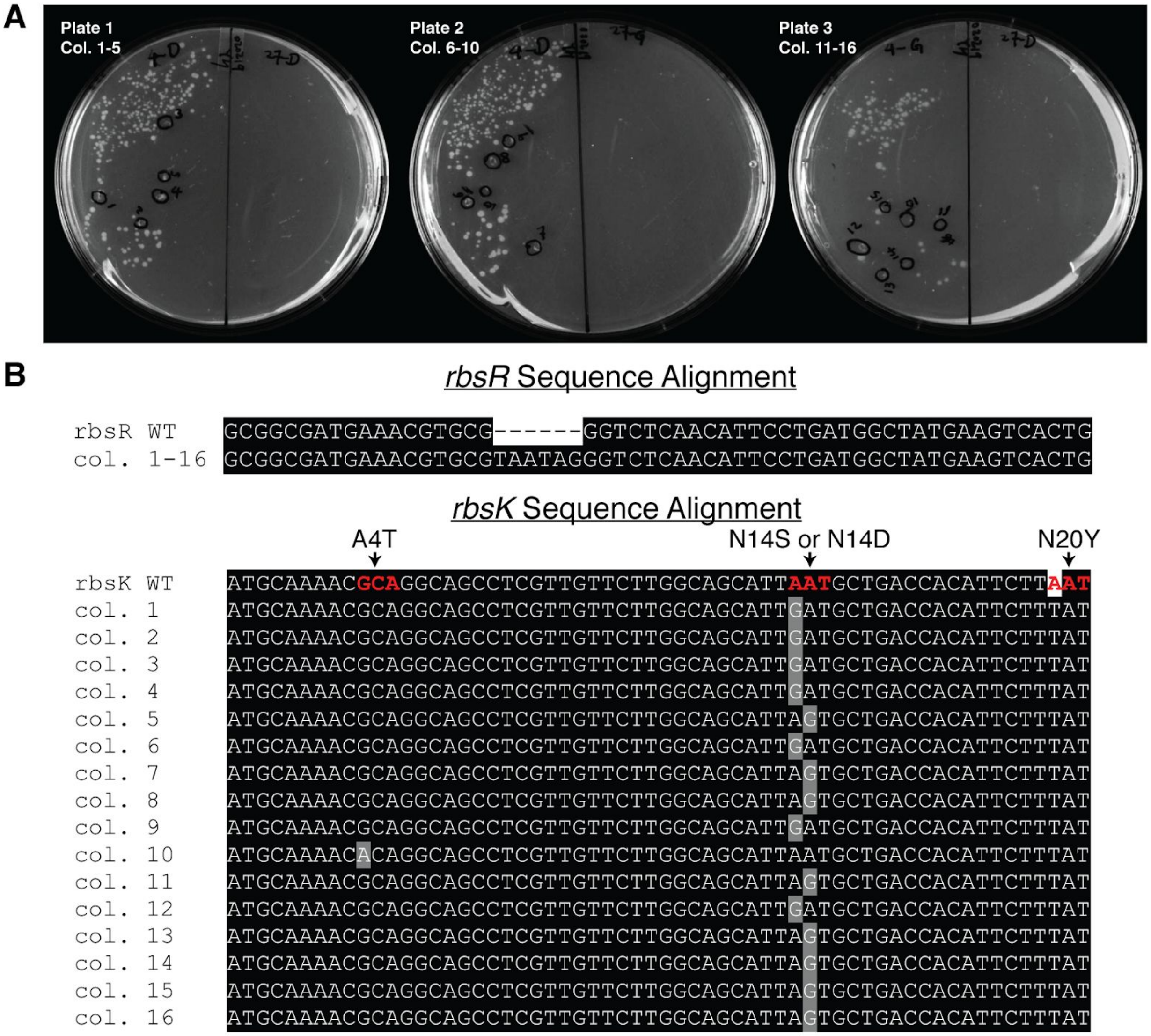

Appendix Figure S4: D-2-deoxyribose pORTMAGE library growth experiments. A) Three D-2-deoxyribose M9 minimal medium agar plates are displayed with a pORTMAGE library plated on the left and the wild type (WT) K-12 MG1655 ancestral strain plated on the right. For the three plates, the growth medium used for the pre-cultures varied. For plate 1, the pORTMAGE library and WT were both grown in D-2-deoxyribose M9 minimal medium, along with some residual LB and glycerol from from the frozen stocks, prior to plating (pre-culture growth on D-2-deoxyribose was presumed possible due to residual glycerol and LB from frozen stocks, thus a washing step was incorporated, see Methods). For plate 2, the pORTMAGE glycerol and LB stock library was grown in D-2-deoxyribose M9 minimal medium and the WT was grown in glycerol M9 minimal medium and washed prior to plating. For plate 3, the same pORTMAGE library was grown in glycerol M9 minimal medium and the glycerol and LB stock WT was grown in D-2-deoxyribose M9 minimal medium prior to plating and washing. After 9-10 days of incubation, significant growth was observed for the pORTMAGE library and no growth was observed for WT similarly in all of the conditions. Thus, there appeared to be no major effect on the outcome of growth in relation to the initial preculture from the glycerol and LB stock. A total of sixteen colonies were picked for colony PCR across the 3 plates and the *rbsR* and *rbsK* regions were sequenced using primers listed in Appendix Table S5. The 16 colonies are circled on the plate images. **B)** Sequence alignment is displayed for regions of interest for the *rbsR* and *rbsK* genes for WT and sixteen colonies (col. 1-16). The corresponding three base pair codons for the RbsK protein residues 4, 14, and 20 are highlighted in red in the rbsK WT sequence for reference. It was observed that all sixteen

sequenced colonies contained both the intended *rbsR* insertion mutation (+TAATAG (619-624/993 nt) resulting in an early stop codon at nucleotide position 622) as well as the RbsK N20Y mutation. Apart from the two expected mutations resulting from the pORTMAGE library construction, a third mutation was observed at RbsK residue Asn14 for 15 of the 16 colonies sequenced or at RbsK residue Ala4 for 1 of the 16 colonies sequenced (colony 10). The mutations at residue 14 resulted in either a RbsK N14D mutation (AAT -> GAT codon change) or RbsK N14S mutation (AAT -> AGT codon change). The mutation at residue 4 resulted in a RbsK A4T mutation (GCA -> ACA codon change).

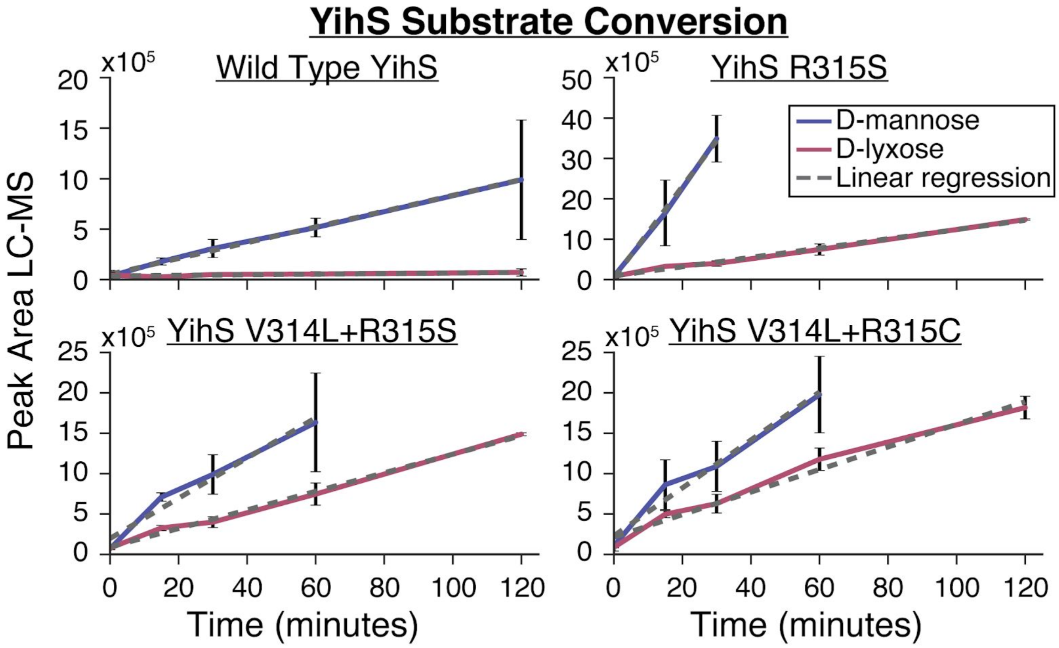

Appendix Figure S5: YihS wild type and mutant substrate conversion. The conversion of D-mannose to D-fructose (blue) and D-lyxose to D-xylulose (red) was monitored using LC-MS. Product formation was tracked over time at a constant enzyme concentration. The cell-free *in vitro* trancription translation system utilized for these assays did not allow for absolute enzyme concentration calculation. Therefore turnover rates on a substrate for a given enzyme cannot be compared to another enzyme; however, the same enzyme concentration was utilized for a given enzyme on both substrates (i.e., the wild type YihS turnover rate on D-mannose observed in these plots cannot be compared to that observed for YihS R315S, but can be compared to the wild type YihS turnover rate on D-lyxose). Turnover rates for each of the above plots were calculated using linear regression (n=3, dotted line). The error bars represent the standard deviation (n=3) of the peak area. The turnover rates could then be used to calculate turnover ratios (Figure 1D).

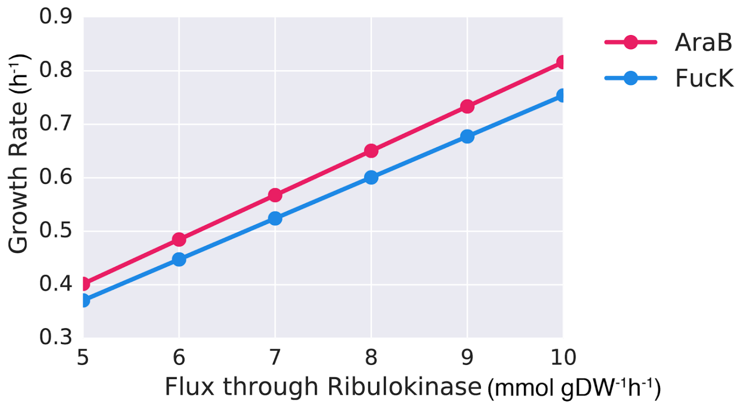

Appendix Figure S6: AraB Ribulokinase activity predicted to improve growth rate *in silico*. Flux balance analysis growth rate predictions as flux is changed through the *fucK*-associated (blue) or *araB*-associated (pink) ribulokinase reaction. The simulations are conducted on models that either contain the FucK or AraB ribulokinase.

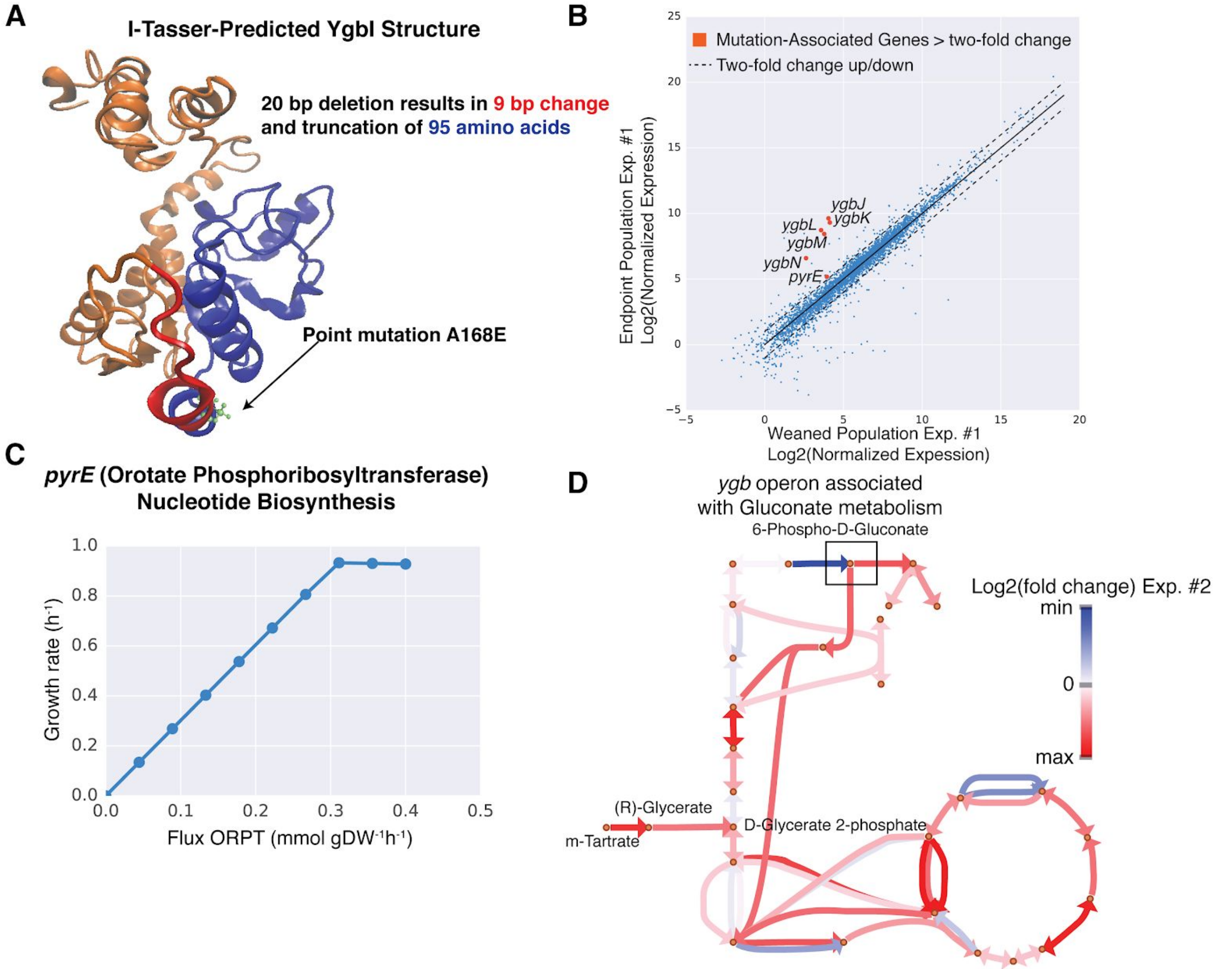

Appendix Figure S7: Optimization mutation analysis for m-tartrate experiments. A) Protein structure prediction of YgbI, the protein associated with a key optimizing mutation. A 20 bp deletion in *ygbI* is predicted to result in a 9 bp change (red) and truncation of 95 amino acids (blue). A point mutation observed should result in a single A168E amino acid change. **B)** RNAseq expression data represented as log2(normalized expression) for duplicates of weaned (beginning of static phase) populations compared to endpoint populations for experiment (Exp.) 1 (n=2 biological replicates for each condition). Ygb genes as well as pyrE, associated with optimizing mutations, are shown to be highly expressed in endpoint populations. **C)** A flux balance analysis plot showing the effect of flux through the reaction associated with *pyrE* on growth rate. Flux through this reaction is predicted to have a significant impact on growth rate. **D)** Log2(fold change) of expression data of Exp. 2 endpoint versus starting point populations (n=2 biological replicates for each condition) superimposed on a pathway map shows increased expression of pathways downstream of 6-phospho-D-gluconate.

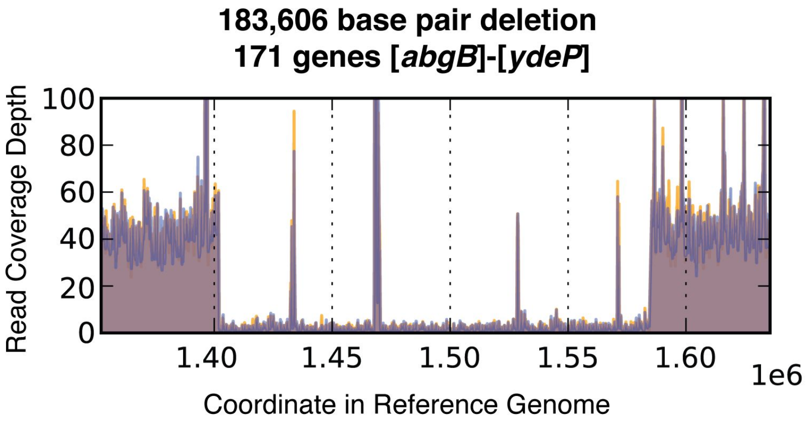

Appendix Figure S8: Large Deletion observed in endpoint population evolved on D-2-deoxyribose. Read depth coverage of the region spanning the 183 kbp deletion observed in the endpoint population grown on D-2-deoxyribose. The spikes in coverage in this region are linked to conserved sequence regions located throughout the genome and can be disregarded as artifacts of these regions of repeating sequences. The genes involved in this deletion were *abgB, abgA, abgR, smrA, ydaM, ydaN, dbpA, ttcA, intR, ydaQ, ydaC, ralR, recT, recE, racC, ydaE, kilR, sieB, ydaF, ydaG, racR, ydaS, ydaT, ydaU, ydaV, ydaW, rzpR, rzoR, trkG, ynaK, ydaY, tmpR, lomR, insH1, lomR, stfR, tfaR, pinR, ynaE, ttcC, uspF, ompN, pfo, ydbJ, hslJ, ldhA, ydbH, ynbE, ydbL, feaR, feaB, tynA, paaZ, paaA, paaB, paaC, paaD, paaE, paaF, paaG, paaH, paaI, paaJ, paaK, paaX, paaY, ynbG, ydbA, insD1, insC1, ydbA, insI1, ydbA, ydbC, ydbD, ynbA, ynbB, ynbC, ynbD, azoR, hrpA, ydcF, aldA, gapC, gapC, cybB, ydcA, hokB, mokB, trg, ydcI, ydcJ, opgD, ydcH, rimL, ydcK, tehA, tehB, ydcL, insP, insQ, ydcO, ydcN, ydcP, yncJ, hicA, hicB, ydcR, ydcS, ydcT, ydcU, ydcV, patD, yncL, ydcX, ydcY, ydcZ, mnaT, curA, mcbR, yncD, yncE, ansP, yncG, yncH, rhsE, ydcD, yncI, yncI, ydcC, pptA, yddH, nhoA, yddE, narV, narW, narY, narZ, narU, yddK, yddK, yddL, yddG, fdnG, fdnH, fdnI, yddM, adhP, maeA, sra, bdm, osmC, ddpF, ddpD, ddpC, ddpB, ddpA, ddpX, dosP, dosC, yddW, gadC, gadB, pqqL, yddB, yddA, ydeM, ydeN, ydeO, safA, ydeP*.

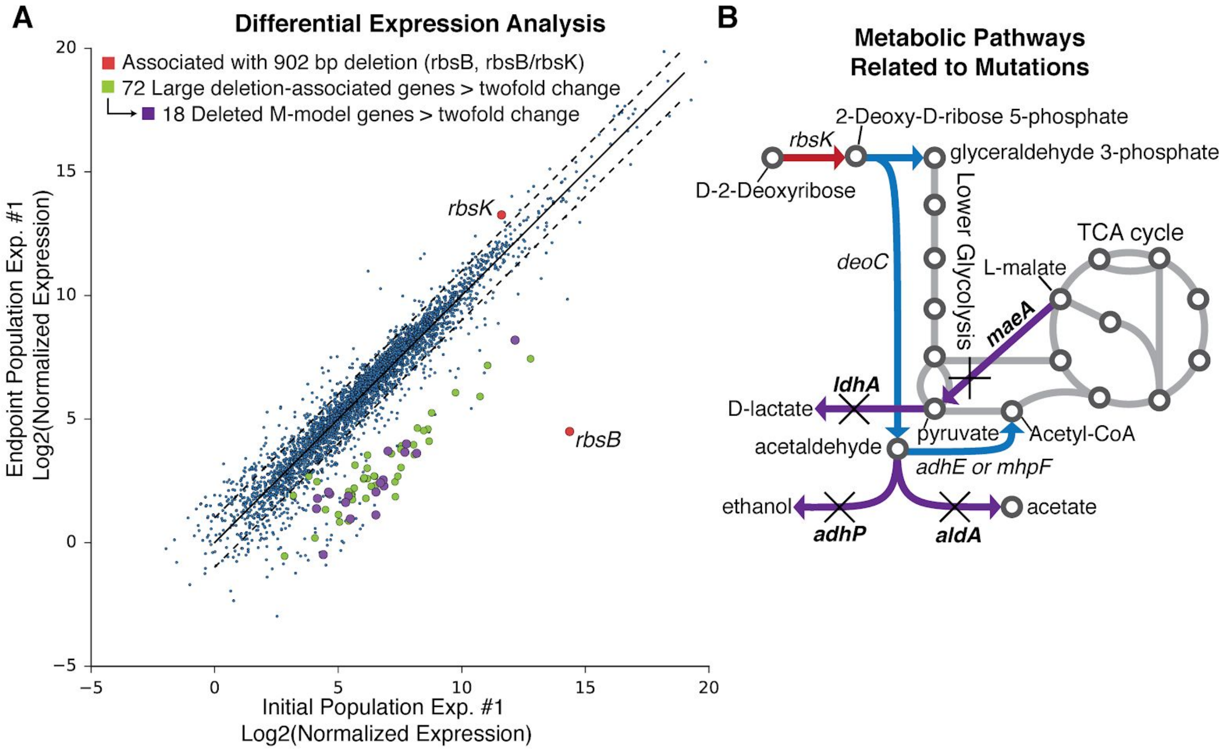

Appendix Figure S9: Deletion mutation analysis for the D-2-deoxyribose experiment. A) RNAseq expression data represented as log2(normalized expression) initial weaned population samples compared to the optimized endpoint population sample for experiment (Exp.) 1. Highlighted in red are rbsB and *rbsK* associated with the 902 base pair deletion event spanning part of the *rbsB* gene and the intergenic region upstream of *rbsK*. Highlighted in green are 72 of the 171 genes located in the large deletion region. These 72 genes were significantly down-regulated and showed more than a 2 fold change. Highlighted in purple are 18 metabolic genes (found in the iJO1366 model) that were significantly down-regulated (Dataset EV3). Flux variability analysis showed that none of the 44 metabolic genes located within the large deletion were necessary to achieve optimal growth (Dataset EV3). The deleted genes have non-zero expression values in the optimized endpoint population, which can be explained by evidence that a fraction of the population does not contain the deletion (Appendix Figure S8). B) A pathway map highlighting predicted pathways for metabolizing D-2-deoxyribose. Starting with D-2-deoxyribose in the upper left, the first reaction is catalyzed by the enzyme associated with the *rbsK* gene noted in red as it was a key gene mutated in the initial weaned population. The following reactions in blue are predicted to feed into lower glycolysis and the TCA cycle. The purple reactions are those linked to metabolic genes that were significantly down-regulated in the endpoint population strain due to the large deletion observed.

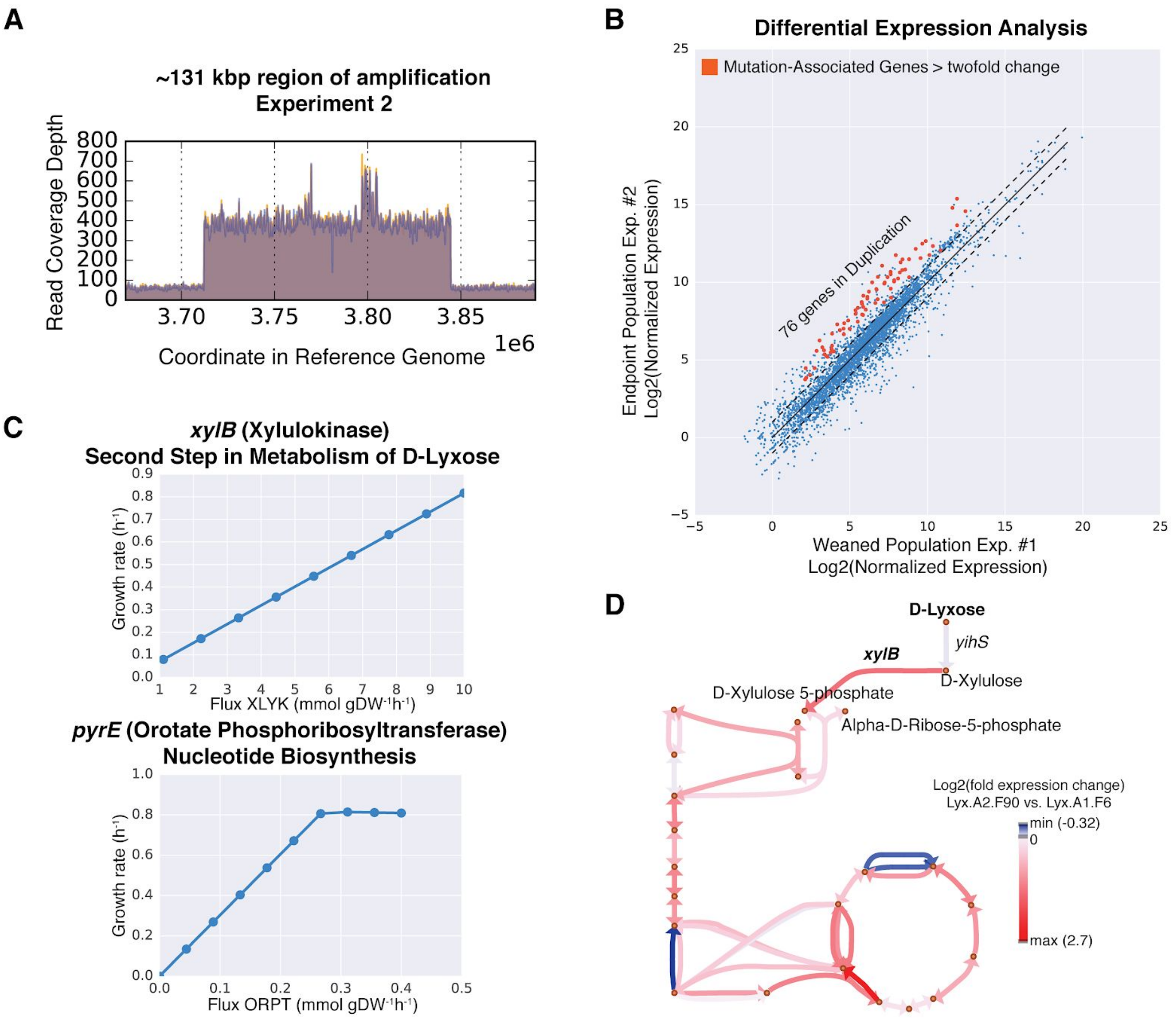

Appendix Figure S10: Optimization mutation analysis for D-lyxose Experiment 2. A) Read depth coverage of the region spanning the 131 kbp region of amplification observed in one of the endpoint populations grown on D-lyxose. The region spans 129 genes (*yhjY-adeD*). **B)** RNAseq expression data represented as log2(normalized expression) for duplicates of the initial populations for experiment 1 compared to the optimized populations for experiment 2 (n=2 biological replicates). Highlighted in red are up-regulated genes associated with the large amplification event. **C)** Two flux balance analysis plots showing the effect of flux through the reactions associated with *xylB* and *pyrE* on growth rate. Flux through these reactions contained within the amplification region are predicted to have a positive effect on growth rate. **D)** A pathway map with log2(fold change) RNAseq data superimposed onto it. The increase in expression of *xylB* as the second step in D-lyxose metabolism is highlighted.

```
RbsK_N20Y_Ecoli      1 MQNAGSLVVLGSINADHILYLQSFPTPGETVTGNHYQVAFGGKGANQAVAAGRSGANIAF
DeoK_Ecoli_p1-3*     1 MD----IAVIGSNMVDLITYTNQMPKEGETLEAPAFKIGCGGKGANQAVAAAKLNSKVLM
DeoK_Styphi          1 MD----IAVIGSNMVDLITYTNQMPKEGETLEAPAFKIGCGGKGANQAVAAAKLNSKVLM

RbsK_N20Y_Ecoli     61 IACTGDDSIGESVRQQLATDNIDITPVSVIKGESTGVALIFVNGEGENVIGIHAGANAAL
DeoK_Ecoli_p1-3*    57 LTKVGDDIFADNTIRNLESWGINTTYVEKVPCTSSGVAPIFVNANSSNSILIIKGANKFL
DeoK_Styphi         57 LTKVGDDIFADNTIRNLESWGINTTYVEKVPCTSSGVAPIFVNANSSNSILIIKGANKFL

RbsK_N20Y_Ecoli    121 SPALVEAQRERIANASALLMQLESPLESVMAAAKIAHQNKTIVALNPAPA-RELPDELLA
DeoK_Ecoli_p1-3*   117 SPEDIDRAAEDLKKCKLIVLQLEVQLETVYHAIEFGKKNGIEVLLNPAPALRELDMSYAC
DeoK_Styphi        117 SPEDIDRAAEDLKKCQLIVLQLEVQLETVYHAIEFGKKHGIEVLLNPAPALRELDMSYAC

RbsK_N20Y_Ecoli    180 LVDIITPNETEAEKLTGIRVENDEDAAKAAQVLHEKGIRTVLITLGSRGVWASVNGEGQR
DeoK_Ecoli_p1-3*   177 KCDFFIPNETELEILTGMSVDTYDHIRLAARSLVDKGLNNIIVTMSEKGALWMTRDQEVH
DeoK_Styphi        177 KCDFFVPNETELEILTGMPVDTYDHIRAAARSLVDKGLNNIIVTMGEKGALWMTRDQEVH

RbsK_N20Y_Ecoli    240 VPGFRVQAVDTIAAGDTFNGALITALLEEKPLPEAIRFAHAAAAIAVTRKGAQPSVPWRE
DeoK_Ecoli_p1-3*   237 VPAFKVNAVDTSGAGDAFIGCFSHYYVQSGDVEAALKKAALFAAFSVTGKGTQSSYPSIE
DeoK_Styphi        237 VPAFRVNAVDTSGAGDAFIGCFAHYYVQSGDVEAAMKKAVLFAAFSVTGKGTQSSYPSIE

RbsK_N20Y_Ecoli    300 EIDAFLDRQR
DeoK_Ecoli_p1-3*   297 QFNEFLTLNE
DeoK_Styphi        297 QFNEYLSLNE
```

**E. coli* strains (AL862, 55989, CT073)[17-19] possessing *deoK* and the ability to grow on D-2-deoxyribose

Appendix Figure S11: Sequence alignment of RbsK mutant and DeoK from pathogenic strains. The sequence alignment of the *E. coli* K-12 Mg1655 RbsK N20Y mutant sequence from this paper, three pathogenic *E. coli* Deok sequence (same for all three), and the Salmonella enterica serovar Typhii DeoK sequence (T-Coffee alignment) is displayed (Bernier-Febreau *et al*, 2004; Monk *et al*, 2013; Tourneux *et al*, 2000). Highlighted in red is the location of the N20Y amino acid substitution that occurred during the evolution experiment on D-2-deoxyribose. This tyrosine residue is shared between all sequences and may be important for binding D-2-deoxyribose.

Supporting Text

<u>*Relevance of observed mutations in the wild*</u>

Were the mutations observed in our laboratory experiment relevant for environmental adaptations in the wild? The N20Y mutation, observed in the RbsK enzyme during the evolution on D-2-deoxyribose, served as a case study. Previous work has found that predominantly intestinal and extraintestinal strains of *E. coli*, as well as some *Salmonella* species, can use D-2-deoxyribose as a sole carbon source as they possess a pathogenicity island containing the deoxyribokinase *deoK* (Bernier-Fébreau *et al*, 2004; Monk *et al*, 2013; Tourneux *et al*, 2000), which shares a 36% sequence identity with *rbsK* (BLASTp (Altschul *et al*, 1997) expect (E) value 4e-29). Specifically, four such reported pathogenic strains in this set (three *E. coli* and one *Salmonella*) (Bernier-Fébreau *et al*, 2004; Monk *et al*, 2013; Tourneux *et al*, 2000) were shown to grow on D-2-deoxyribose and possess a deoxyribokinase (DeoK) with a tyrosine residue at the equivalent N20Y position (Appendix Figure S11). This information suggests that the N20Y mutation may have improved the ribokinase underground activity of RbsK in the mutant strain evolved here on D-2-deoxyribose and enabled growth in this environment similar to the capabilities of the strains that possess the DeoK enzyme. Therefore, this case highlighted that the genetic basis of adaptation observed in the laboratory may indeed be relevant to evolution in the wild; however, this is a single example and generating many more replicates of this experiment (or the other substate evolutions) could better determine if relevance to adaptation outside of the lab.

<u>*M-tartrate optimizing mutations*</u>

Important mutations identified for optimizing growth on m-tartrate were linked to *ygb* operon genes, mostly uncharacterized y-genes, as well as *pyrE*, associated with nucleotide biosynthesis. Mutations in *ygbI*, a putative DNA-binding transcriptional regulator, were observed in both independent replicate evolution experiments (Table 2, Appendix Figure S7). Not much information is available about this putative transcriptional regulator; however, mutations highlighted on an I-TASSER-predicted(Yang *et al*, 2015) structure coupled with analysis of the expression levels of other *ygb* genes suggest that *ygbI* acts as a repressor for the transcription of *ygb* genes. In particular, the 20 base pair deletion observed in the endpoint population of experiment 2 should result in a removal of 95 amino acids of the YgbI protein (Appendix Figure S7). Such a large truncation of the protein would likely lead to a loss of function of YgbI, thereby increasing expression of *ygb* operon genes if the YgbI regulator acts as a repressor. A mutation in *ygbI* was acquired early in the experiment 2 population as evidenced by the clonal resequencing data (Datasets EV1, EV2). RNAseq expression data for experiment 2 initial and endpoint populations showed that *ygbIJKLMN* genes were highly expressed in both the initial and endpoint populations, whereas for experiment 1, only the endpoint population containing the *ygbI* SNP mutation showed high expression of the *ygb* operon genes (Appendix Figure S7). Not much is known about the *ygb* genes, however, previous work in the soft-rot pathogen *Pectobacterium carotovorum* has linked it to gluconate metabolism(Mole *et al*, 2010). Examination of the log2 fold change values of expression data for experiment 2 overlaid on a metabolic map showed that pathways branching from

6-phospho-D-gluconate were highly expressed (Appendix Figure S7), possibly suggesting that the *ygb* genes are somehow involved in converting m-tartrate catabolism-associated metabolites to gluconate; however, this hypothesis is yet to be examined experimentally.

The second set of optimizing mutations believed to aid in increasing the growth rate of the evolved populations on m-tartrate were linked to the expression of *pyrE*. Sequencing data of both endpoint populations showed mutations in the intergenic region between *rph* and *pyrE*. Expression data for both evolution experiments showed that *pyrE* was significantly up-regulated in the endpoint populations compared to the initial populations (Appendix Figure S7B). Flux balance analysis examining the effect of flux through the *pyrE*-associated reaction (orotate phosphoribosyl transferase) revealed that this reaction does play an important role in the growth of strains on m-tartrate. Increasing flux through the reaction results in an increased growth rate. Thus, enhanced growth on m-tartrate appears to be influenced by expression of *ygb* genes as well as *pyrE*.

References:

Altschul SF, Madden TL, Schäffer AA, Zhang J, Zhang Z, Miller W & Lipman DJ (1997) Gapped BLAST and PSI-BLAST: a new generation of protein database search programs. *Nucleic Acids Res.* **25:** 3389–3402

Bernier-Febreau C, du Merle L, Turlin E, Labas V, Ordonez J, A.-M. G & Le Bouguenec* C (2004) Use of Deoxyribose by Intestinal and Extraintestinal Pathogenic Escherichia coli Strains: a Metabolic Adaptation Involved in Competitiveness. *Infect. Immun.* **72:** 7381–7381

Bernier-Fébreau C, du Merle L, Turlin E, Labas V, Ordonez J, Gilles A-M & Le Bouguénec* C (2004) Use of Deoxyribose by Intestinal and Extraintestinal Pathogenic Escherichia coli Strains: a Metabolic Adaptation Involved in Competitiveness. *Infect. Immun.* **72:** 7381–7381

LeBlanc DJ & Mortlock RP (1971) Metabolism of D-arabinose: origin of a D-ribulokinase activity in Escherichia coli. *J. Bacteriol.* **106:** 82–89

Mole B, Beth M, Sohrab H, Dangl JL & Grant SR (2010) Gluconate Metabolism Is Required for Virulence of the Soft-Rot Pathogen Pectobacterium carotovorum. *Mol. Plant. Microbe. Interact.* **23:** 1335–1344

Monk JM, Charusanti P, Aziz RK, Lerman JA, Premyodhin N, Orth JD, Feist AM & Palsson BØ (2013) Genome-scale metabolic reconstructions of multiple Escherichia coli strains highlight strain-specific adaptations to nutritional environments. *Proc. Natl. Acad. Sci. U. S. A.* **110:** 20338–20343

Notebaart RA, Szappanos B, Kintses B, Pál F, Györkei Á, Bogos B, Lázár V, Spohn R, Csörgő B, Wagner A, Ruppin E, Pál C & Papp B (2014) Network-level architecture and the evolutionary potential of underground metabolism. *Proc. Natl. Acad. Sci. U. S. A.* **111:** 11762–11767

Podolny V, Lin EC & Hochschild A (1999) A cyclic AMP receptor protein mutant that

constitutively activates an Escherichia coli promoter disrupted by an IS5 insertion. *J. Bacteriol.* **181:** 7457–7463

Tourneux L, Bucurenci N, Saveanu C, Kaminski PA, Bouzon M, Pistotnik E, Namane A, Marlière P, Bârzu O, Li De La Sierra I, Neuhard J & Gilles AM (2000) Genetic and biochemical characterization of Salmonella enterica serovar typhi deoxyribokinase. *J. Bacteriol.* **182:** 869–873

Yang J, Yan R, Roy A, Xu D, Poisson J & Zhang Y (2015) The I-TASSER Suite: protein structure and function prediction. *Nat. Methods* **12:** 7–8
